# Age-Related Differences in Structure and Function of Nasal Epithelial Cultures From Healthy Children and Elderly People

**DOI:** 10.3389/fimmu.2022.822437

**Published:** 2022-02-28

**Authors:** Anita Balázs, Pamela Millar-Büchner, Michael Mülleder, Vadim Farztdinov, Lukasz Szyrwiel, Annalisa Addante, Aditi Kuppe, Tihomir Rubil, Marika Drescher, Kathrin Seidel, Sebastian Stricker, Roland Eils, Irina Lehmann, Birgit Sawitzki, Jobst Röhmel, Markus Ralser, Marcus A. Mall

**Affiliations:** ^1^Department of Pediatric Respiratory Medicine, Immunology and Critical Care Medicine, Chariteí - Universitaätsmedizin Berlin, Berlin, Germany; ^2^German Center for Lung Research (DZL), Associated Partner Site, Berlin, Germany; ^3^Charité - Universitätsmedizin Berlin, Core Facility - High-Throughput Mass Spectrometry, Berlin, Germany; ^4^Charité - Universitätsmedizin Berlin, Department of Biochemistry, Berlin, Germany; ^5^Center for Digital Health, Berlin Institute of Health at Charité - Universitätsmedizin Berlin, Berlin, Germany; ^6^Molecular Epidemiology Unit, Berlin Institute of Health at Charité - Universitätsmedizin Berlin, Berlin, Germany; ^7^Institute of Medical Immunology, Charité - Universitätsmedizin Berlin, Berlin, Germany; ^8^The Francis Crick Institute, Molecular Biology of Metabolism Laboratory, London, United Kingdom; ^9^Berlin Institute of Health (BIH) at Charité, Berlin, Germany

**Keywords:** primary nasal epithelial cultures, aging, airways disease, ion transport, proteome

## Abstract

The nasal epithelium represents the first line of defense against inhaled pathogens, allergens, and irritants and plays a key role in the pathogenesis of a spectrum of acute and chronic airways diseases. Despite age-dependent clinical phenotypes triggered by these noxious stimuli, little is known about how aging affects the structure and function of the airway epithelium that is crucial for lung homeostasis and host defense. The aim of this study was therefore to determine age-related differences in structural and functional properties of primary nasal epithelial cultures from healthy children and non-smoking elderly people. To achieve this goal, highly differentiated nasal epithelial cultures were established from nasal brushes at air–liquid interface and used to study epithelial cell type composition, mucin (MUC5AC and MUC5B) expression, and ion transport properties. Furthermore, we determined age-dependent molecular signatures using global proteomic analysis. We found lower numeric densities of ciliated cells and higher levels of MUC5AC expression in cultures from children vs. elderly people. Bioelectric studies showed no differences in basal ion transport properties, ENaC-mediated sodium absorption, or CFTR-mediated chloride transport, but detected decreased calcium-activated TMEM16A-mediated chloride secretory responses in cultures from children vs. elderly people. Proteome analysis identified distinct age-dependent molecular signatures associated with ciliation and mucin biosynthesis, as well as other pathways implicated in aging. Our data identified intrinsic, age-related differences in structure and function of the nasal epithelium and provide a basis for further studies on the role of these findings in age-dependent airways disease phenotypes observed with a spectrum of respiratory infections and other noxious stimuli.

## Introduction

The airway mucosa represents the first line of defense of the respiratory system against pathogens, pollutants, and irritants that are constantly inhaled during tidal breathing. At the interface with the environment, airway epithelial cells have developed specialized functions to provide host protection, such as barrier function, secretion of anti-microbial mediators, interaction with cells of the immune system, as well as elimination of potentially harmful stimuli by mucociliary clearance (MCC) (1, 2). MCC operates through the coordinated function of (i) the motile cilia, (ii) the airway surface liquid layer, and (iii) the mucus layer (3–6). Airway mucus is a viscoelastic hydrogel composed of ~97% water and ~3% solids, including the highly glycosylated polymeric mucin glycoproteins MUC5AC and MUC5B, as well as salts, lipids, and anti-microbial peptides (7). Hydration and transportability of mucus are critically dependent on ion and fluid transport across the airway epithelium, which is primarily determined by the activity of sodium absorption through the epithelial sodium channel (ENaC) and chloride secretion through the cyclic adenosine monophosphate (cAMP)-regulated chloride channel cystic fibrosis transmembrane conductance regulator (CFTR), as well as the calcium activated chloride channel transmembrane protein 16A (TMEM16A) (8, 9). In health, a well-hydrated mucus layer is continuously transported by directional beating of the cilia towards the throat, providing effective elimination of mucus entrapped particles (10, 11). Proper MCC is crucial for airway homeostasis, and mucociliary dysfunction has been implicated in the pathogenesis of acute and chronic airways diseases caused by a spectrum of pathogens, allergens, and other environmental pollutants (4, 12). Of note, the clinical airways disease phenotypes triggered by some of these noxious stimuli are strikingly age-dependent, suggesting a potential role of age-related differences in airway epithelial defense properties (13–20). However, the relationship between age and airway epithelial structure and function has not been studied.

*In vitro* studies utilizing highly differentiated primary human airway epithelial cell cultures grown at air–liquid interface (ALI) provided an essential contribution to our current understanding of airway epithelial innate defense (21). ALI cultures recapitulate key physiological features of the airways *in vivo* including the pseudostratified morphology, composition of relevant proportions of airway epithelial cell types, barrier function, coordinated ciliary beating, and mucus secretion (22–24). The emergence of improved cell expansion protocols enabled the generation of sufficient cell numbers to differentiate cultures from nasal brushings that can be obtained non-invasively (25). The ALI culture system has been instrumental to study genetically determined airways diseases such as cystic fibrosis or primary ciliary dyskinesia (26, 27). Furthermore, a number of studies demonstrated that ALI cultures retain donor-dependent phenotypic characteristics such as signatures of tobacco smoking, inflammation, or even aging, suggesting that epigenetic factors may remain stable even through rounds of proliferative expansion (28–31).

The aim of this study was to determine the effects of aging on the structure and function of the nasal epithelium. To achieve this goal, we generated highly differentiated nasal epithelial ALI cultures from healthy children and non-smoking elderly people and compared cell-type composition, mucin (MUC5AC and MUC5B) expression, transepithelial ion transport properties, and global proteome changes between these age groups.

## Materials and Methods

### Study Population

This study was conducted in accordance with the Declaration of Helsinki and approved by the ethics committee at the Charité-Universitätsmedizin Berlin (EA2/066/20). Written informed consent was obtained from all study participants, their parents, or legal guardians. In total, our study included nasal swabs from 17 healthy children (≤10 years old) and 14 healthy non-smoking elderly people (≥60 years old). Demographics and clinical characteristics of the study population are provided in **Table 1**.

**Table 1:** Demographics of the study population.

	Children	Elderly
Number (*n*)	17	14
Mean age (years ± SD)	4.9 ± 3.1	73.1 ± 9.4
Sex (% male)	76.5	50.0
Smoker (*n*)	0	0
Asthma (*n*)	0	0
Allergy (*n*)*	0	3

*Allergy to house dust mite or pollen.

### Culture of Primary Human Nasal Epithelial Cells

Primary human nasal epithelial cells were obtained by nasal brushings. Cultivation of cells was performed by the conditionally reprogrammed cell culture method as previously described (25). In brief, brushed cells were expanded in co-culture with irradiated mouse 3T3 fibroblasts in the presence of RhoA kinase inhibitor Y-27632. Epithelial cells were seeded at passage 2 or 3 on human placental type IV collagen–coated, 0.4-mm pore size Snapwell or Transwell 1.1 cm^2^ supports (Corning, Glendale, NY, USA) at a density of 200,000 cells/cm^2^ in UNC-ALI medium and differentiated at ALI for at least 4 weeks. Cultures were used for analysis when transepithelial electrical resistance (TEER) was ≥500 Ω*cm^2^.

### Immunostaining

Cultures were first washed with PBS, then fixed by 4% paraformaldehyde for 10 min, and permeabilized with 0.1% Triton X-100 for 8 min and blocked with 5% goat serum for 30 min. The primary antibodies used were rat monoclonal anti-α-tubulin (mAb1864, Millipore, Burlington, MA, USA), mouse monoclonal anti-MUC5AC (sc-59951, Santa Cruz, Dallas, TX, USA), and rabbit polyclonal anti-KRT5 (SAB1410739, Sigma, St. Louis, MO, USA) at dilution of 1:200 for 1 h. For TMEM16A localization, rabbit polyclonal anti-TMEM16A antibody (HPA032148, Atlas Antibodies, Stockholm, Sweden) was used at a dilution of 1:50 overnight at 4°C. The secondary antibodies used were Alexa Fluor 488-conjugated goat anti-rat IgG (SA5-10018, Thermo Fisher Scientific, Waltham, MA, USA), Alexa Fluor 647-conjugated goat anti-mouse IgG (A-21235, Thermo Fisher Scientific, Waltham, MA, USA), and Alexa Fluor 750-conjugated goat anti-rabbit IgG (A-21039 Thermo Fisher Scientific, Waltham, MA, USA) at 1:300 dilution for 30 min. Zonula occludens (ZO-1) antibody conjugated with Alexa Fluor 555 (MA3-39100-A555, Thermo Fisher Scientific, Waltham, MA, USA) and Hoechst 33342 (Thermo Fisher Scientific, Waltham, MA, USA) was incubated for 30 min at 1:300 and 1:5,000 dilution, respectively. Filters were cut out with a scalpel and mounted with ProLong™ Gold anti-fade reagent (Thermo Fisher Scientific, Waltham, MA, USA). All steps were performed at room temperature, unless indicated otherwise. Images were acquired using a Leica Stellaris 8 confocal laser scanning microscope equipped with Hamamatsu Orca Flash 4.0 V3 sCMOS camera for wide-field fluorescence imaging.

### Quantification of Cell Types

Due to the heterogeneity of ciliated cell distribution within one filter, tile scans of the whole filter area were acquired and stitched together in a single image (~40 mm^2^ filter area on average). The cultures were imaged with 10× air objective in wide-field mode (α-tubulin^+^ cells) or in confocal mode with opened pin-hole (KRT5^+^ or MUC5AC^+^ cells). Images were analyzed with FIJI software (32). Cells with positive signal (α-tubulin^+^, MUC5AC^+^ or KRT5^+^) were segmented by creating a binary mask with the application of an intensity threshold, where over/under-saturated pixels were adjusted based on visual control of the original image. Pixels were dilated and overlapping objects were separated by watershed command. Particles between 20 and 150 µm^2^ were analyzed and cell counts per surface area were determined. Representative stacks were acquired with 20× immersion objective in confocal mode with Lightning module and maximal Z-projections are shown.

### RNA Extraction and RT-PCR

Total RNA was isolated using RNeasy Micro Kit (Qiagen, Hilden, Germany) according to the manufacturer’s instructions. RNA was transcribed by high-capacity cDNA reverse transcription kit (Applied Biosystems, Darmstadt, Germany). Real-time PCR was performed using Applied Biosystems 7500 Real-Time PCR system with TaqMan Universal PCR master mix and inventoried TaqMan gene expression assays (Applied Biosystems, Darmstadt, Germany) for human *CFTR* (Hs00357011_m1), *TMEM16A* (Hs00216121_M1), *MUC5AC* (Hs01365616_m1), *MUC5B* (Hs00861595_m1), and *ACTB* (4333762F). The relative expression ratios were calculated from the RT-PCR efficiencies and the crossing point deviation of target gene transcripts in comparison to the reference gene transcript *ACTB* (33).

### Preparation of Cell Lysates

Filters were washed with PBS and 80 µl of RIPA buffer (Thermo Fisher Scientific, Waltham, MA, USA) containing cOmplete protease inhibitor (Merck, Darmstadt, Germany) was added; the cells were scraped and vortexed briefly. Three filters/individual were pooled, and samples from different individuals were considered as biological replicates. After 30-min incubation on ice, the lysates were cleared by centrifugation. The protein concentration of the supernatant was determined using Pierce™ BCA Protein Assay Kit (Thermo Fisher Scientific, Waltham, MA, USA), according to the manufacturer’s instructions.

### Mucin Agarose Gel Electrophoresis

Mucin Western blot was performed as previously described (34). In brief, 36 µg of total protein was loaded in equal volume of 30 µl. Agarose gel electrophoresis using 0.8% agarose was combined with transfer onto a nitrocellulose membrane *via* vacuum. After loading the gels, proteins were separated on 0.8% agarose gel at 80 V (1 h) with Tris-acetate-EDTA/SDS buffer. For an efficient mucin transfer, the gel was reduced for 20 min in a solution containing 10 mM dithiothreitol (DTT) and proteins were then transferred by vacuum blotting (MP Biomedicals, Irvine, CA, USA) to nitrocellulose membranes. For total protein normalization, Ponceau S (Advansta, San Jose, CA, USA) staining was used. Blots were probed with mouse monoclonal antibodies against MUC5B (sc-393952, Santa Cruz, Dallas, TX, USA) and MUC5AC (MA5-12178, Invitrogen, Waltham, MA, USA). Primary antibodies were diluted 1:250 in 1% milk-PBS. The secondary antibody was goat anti-mouse immunoglobulins/HRP (P0047, Dako, Glostrup, Denmark), diluted 1:2,000 in 1% milk-PBS. Restore™ PLUS Western Blot Stripping Buffer (Thermo Fisher Scientific, Waltham, MA, USA) was used for membrane stripping according to the manufacturer’s instructions. Pierce™ ECL Western Blotting-Substrate (Thermo Fisher Scientific, Waltham, MA, USA) in combination with ChemiDoc Imaging System (Bio-Rad, Hercules, CA, USA) were used for the detection. Densitometric analysis was performed by FIJI software (32).

### Ussing Chamber Experiments

Transepithelial ion transport experiments were performed in EasyMount Ussing chambers (Physiologic Instruments, San Diego, CA, USA) using voltage clamp configuration to measure the short-circuit current (I_sc_). The I_sc_ was continuously recorded using Lab-Chart8 (AF Instruments, Oxfordshire, UK), and transepithelial resistance was monitored by application of short voltage pulses (2 mV) every 60 s. Experiments were performed under chloride gradient conditions (basolateral 145 mM vs. apical 5 mM) to increase the electrochemical driving force for chloride secretion and augment chloride secretory responses across the epithelium as previously described (24, 35, 36). After 15–20 min equilibration, basal I_sc_ was measured and amiloride (100 µM) was added to inhibit sodium absorption *via* ENaC. Next, forskolin (Fsk, 10 µM) and 3-isobutyl-1-methylxanthin (IBMX, 100 µM) were added together, followed by CFTR-inhibitor 172 (CFTRinh172, 20 µM) to assess CFTR-mediated chloride secretion. Uridine-triphosphate (UTP, 10 µM) was added to evaluate the calcium-activated chloride secretion. In a subset of experiments, UTP was followed by small molecular weight TMEM16A inhibitor Ani9 (10 µM).

### Sample Preparation for Proteomic Analysis

One hundred micrograms of protein was transferred to AFA tubes (PN 520292, 500639) and filled to 60 µl with RIPA buffer. Proteins were extracted and DNA sheared DNA (Covaris LE220Rsc: PIP 350 W, DF 25%, CPB 200, 2 repeats, 300 s pulse, 20 C). Protein (25 µg) was used for SP3 protein preparation on a Biomek i7 workstation with single-step reduction and alkylation (37). Briefly, 16.6 μl reduction and alkylation buffer (40 mM TCEP, 160 mM CAA, and 200 mM ABC) were added, and samples were incubated for 5 min at 95°C and cooled to room temperature. Proteins were bound to 2.5 μg of paramagnetic beads (1:1 ratio of hydrophilic/hydrophobic beads) by adding acetonitrile (ACN) to 50%. Samples were washed twice with 80% ethanol and once with 100% ACN, before reconstitution in 35 μl of 100 mM ABC. Digestion was completed overnight at 37°C using a trypsin/LysC enzyme mix (Promega, Madison, WI, USA) at a protein:enzyme ratio of 50:1 (w/w) and stopped with formic acid (0.1%). The peptides were stored at −80°C until analysis by LC-MS/MS without further conditioning or clean-up.

### Liquid Chromatography Mass Spectrometry

The tryptic digests were injected on the 25-cm Aurora Series with emitter column (CSI, 25 cm × 75 µm ID, 1.6 µm C18, IonOpticks), installed in the nano-electrospray source (CaptiveSpray source, Bruker Daltonics, Germany) at 50°C using UltiMate 3000 (Thermo Scientific Dionex) coupled with TIMS quadrupole time-of-flight instrument (timsTOF Pro2, Bruker Daltonics, Germany) and measured in diaPASEF mode (38). The mobile phases water/0.1% FA and ACN/0.1% FA (A and B, respectively) were applied in linear gradients starting from 2% B and increasing to 17% in 87 min, followed by an increase to 25% B in 93 min, 37% B in 98 min, and 80% B in 99 min to 104 min; the column was equilibrated in 2% B in the next 15 min. For calibration of ion mobility dimension, three ions of Agilent ESI-Low Tuning Mix ions were selected (m/z [Th], 1/K0 [Th]: 622.0289, 0.9848; 922.0097, 1.1895; 1221.9906, 1.3820). The diaPASEF windows scheme was ranging in dimension m/z from 396 to 1,103 Th and in dimension 1/K0 0.7–1.3 Vs/cm^2^, with 59 × 12 Th windows). All measurements were done in low sample amount mode with ramp time 166 ms.

### Protein Identification and Quantification

The raw data were processed using DIA-NN 1.8 (39) with the ion mobility module for diaPASEF (40). MS2 and MS1 mass accuracies were both set to 10 ppm, and scan window size was set to 10. DIA-NN was run in library-free mode with standard settings (fasta digest and deep learning-based spectra, RT and IMs prediction) using the uniprot human reference proteome annotations (41) (downloaded on 2019.12.20) and the match-between-runs (MBR) option.

### Proteomics Data Processing, and Statistical and Functional Analysis

Peptide normalized intensities were subjected to quality control with all 27 samples passing acceptance criteria. Peptides with excessive missing values (>35% per group) were excluded from analysis. The missing values of the remaining peptides were imputed group-based using the PCA method (42). Normalization was performed with LIMMA (43) implementation of cyclic loess method (44) with option “fast” (45). To obtain a quantitative protein data matrix, the log_2_ intensities of peptides were filtered, and only peptides belonging to one protein group were kept and then summarized into protein log intensity by the “maxLFQ” method (46), implemented in R package iq (47). Sample protein distributions were median centered. Statistical analysis of proteomics data was carried out using internally developed R scripts based on publicly available packages. PCA exploratory analysis was carried out using the R package FactoMineR (48). Linear modeling was based on the R package LIMMA (43). The following model was applied to each tissue dataset (log(p) is log_2_ transformed expression of a protein): log(p) ~ 0 + Class. The categorical factor Class had two levels: old, young; reference level: young. To find regulated features, the following criteria were applied: Significance level alpha was set to guarantee the false discovery rate below ~5%. We found that alpha = 0.005 was delivering the required level of Benjamini–Hochberg FDR (49). The log fold-change criterion was applied to guarantee that the measured signal is above the average noise level. As such, we have taken median residual standard deviation of linear model: log_2_ (T) = median residual SD of linear modeling (= log_2_(1.38)). Functional analysis was carried out using the R package clusterProfiler (50) for GSEA. Log_2_ fold-changes old/young of all quantified proteins were used for their ranking and calculation of the enrichment score. For selecting the most (de)regulated GO terms, we applied the following filter: 5 ≤ term size ≤ 350. Unless specified separately, analyses were carried out with Benjamini–Hochberg FDR threshold 5%. To identify epithelial cell subtypes from our proteome data, we extracted cell-type markers from the Single-cell atlas of the airway epithelium scRNAseq dataset (grch38 genes annotation) and associated the log_2_ fold-change proteome values (51).

### Statistics

Data were analyzed with GraphPad Prism 9.1.2 for Windows (GraphPad Software, San Diego, CA, USA) and are reported as mean ± standard error of the mean (SEM). Two group comparisons were performed with Student’s t-test or Mann–Whitney Rank Sum test as appropriate. *p* < 0.05 was accepted to indicate statistical significance.

## Results

### Nasal Epithelial Cultures From Children Display Lower Numbers of Ciliated Cells, Higher Numbers of MUC5AC^+^ Secretory Cells, and Elevated MUC5AC Expression

**Figure 1:**
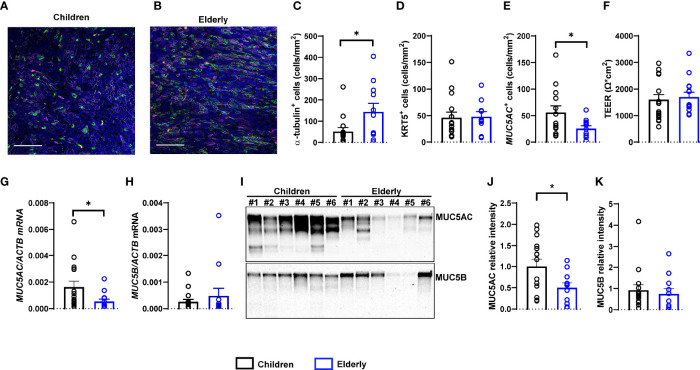
Age-related differences in numeric densities of ciliated cells and MUC5AC expression in nasal epithelial cultures from children compared to elderly people. **(A, B)** Representative images of immunofluorescence of nasal epithelial cultures from healthy children and elderly people. Green: α-tubulin (ciliated cells), magenta: KRT5 (basal cells), yellow: MUC5AC (secretory cells), white: ZO-1 (tight junctions), and blue: Hoechst (cell nuclei). Scale bar, 100 µm. **(C–E)** Quantification of α-tubulin^+^ (ciliated) cells **(C)**, KRT5^+^ (basal) cells **(D)**, and MUC5AC^+^ (secretory) cells **(E)** (*n* = 14 and 11 individuals per group). **(F)** Transepithelial electrical resistance (TEER) (*n* = 17 and 14 individuals per group). **(G, H)** Transcript levels of *MUC5AC* **(G)** and *MUC5B* **(H)** (*n* = 16 and 12 individuals per group). **(I)** Representative MUC5AC and MUC5B Western blot. **(J, K)** Protein quantification of MUC5AC **(J)** and MUC5B **(K)** by densitometry (*n* = 15 and 11 individuals per group). *p < 0.05 compared to children. Data are shown as mean ± S.E.M. Statistical analysis was performed with unpaired two-tailed *t* test in **(D-F, J)**, and with two-tailed Mann–Whitney test in **(C, G, H, K)**.

To investigate potential morphological differences between nasal epithelia of children and elderly people, we established highly differentiated primary cultures at ALI. Cultures were fully differentiated from week 4 onwards and displayed the expected pseudostratified morphology of the respiratory epithelium with visible ciliary beating and mucus secretion. To characterize cell-type composition, we performed whole-mount immuno-histochemistry and quantified ciliated cells (α-tubulin^+^), secretory cells (MUC5AC^+^), and basal cells (KRT5^+^) (**Figures 1A–E**). We found a lower number of ciliated cells in cultures from children compared to elderly people and higher number of MUC5AC^+^ cells, while the number of basal cells did not differ between age groups (**Figures 1C–E**). TEER values were measured as indicators of cell confluence and quality control and were comparable in both age groups (**Figure 1F**). Furthermore, we investigated the transcript and protein levels of the two major secreted mucins MUC5AC and MUC5B. We found that MUC5AC expression was higher both at the transcript and protein level in cultures from children vs. elderly people (**Figures 1G, I, J**). MUC5B expression levels were comparable between age groups, both by RT-PCR and by Western blot analysis (**Figures 1H, I, K**).

### TMEM16A-Mediated Chloride Secretion Is Decreased in Nasal Epithelial Cultures From Children

**Figure 2:**
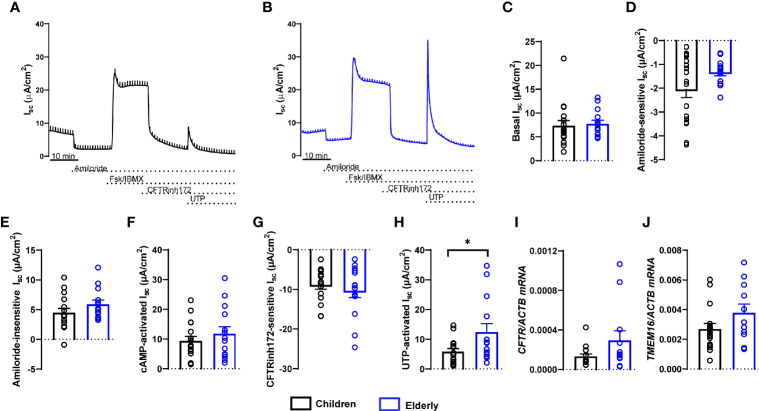
Age-related differences in calcium-activated chloride secretion in nasal epithelial cultures from healthy children compared to elderly people. **(A, B)** Representative original recordings of transepithelial Ussing chamber measurements in primary nasal epithelial cultures from children and elderly people. **(C–G)** Summary of individual effects of basal I_sc_ **(C)**, amiloride-sensitive I_sc_ **(D)**, amiloride-insensitive I_sc_ **(E)**, cAMP-activated I_sc_ **(F)**, CFTR inhibitor 172-sensitive I_sc_ **(G)**, and UTP-activated I_sc_ **(H)** (*n* = 17 and 14 individuals per group, data represent mean values of 2–3 filters per individual). **(I, J)** Transcript levels of *CFTR* **(I)** and *TMEM16A* **(J)** (*n* = 16 and 12 individuals per group). *p < 0.05 compared to children. Data are shown as mean ± S.E.M. Statistical analysis was performed with unpaired two-tailed *t*-test in **(D–G)**, and with two-tailed Mann–Whitney test in **(C, H–J)**.

**Figure 3:**
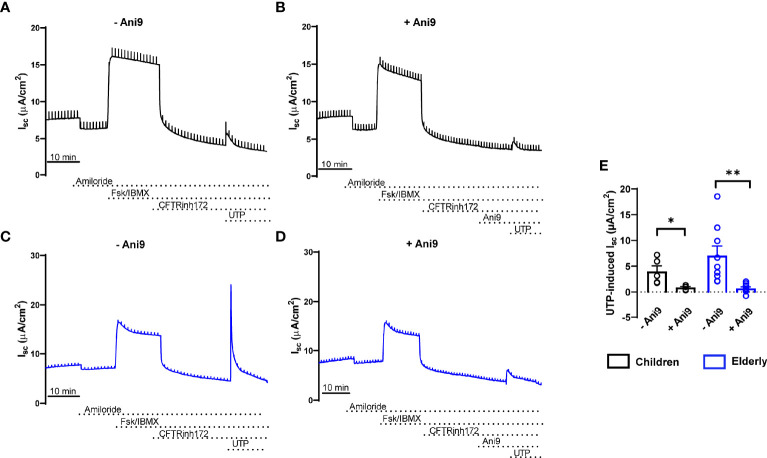
Age-related differences in calcium-activated chloride secretion in nasal epithelial cultures are mediated by TMEM16A. **(A–E)** Representative original recordings and summary data of transepithelial Ussing chamber measurements in primary nasal epithelial cultures from healthy children **(A, B, E)** and elderly people **(C–E)** showing the effect of UTP-induced I_sc_ in the absence **(A, C, E)** and presence **(B, D, E)** of the TMEM16A inhibitor Ani9 (*n* = 4 and 9 individuals per group, data represent mean values of 2–3 filters per individual). *p < 0.05 and **p < 0.01 compared to Ani9- group. Data are shown as mean ± S.E.M. Statistical analysis was performed with paired two-tailed *t* test in **(E)**.

To compare bioelectrical properties of cultures from healthy children and elderly people, we performed transepithelial ion transport measurements in Ussing chambers (**Figures 2A, B**). Basal I_sc_, amiloride-insensitive I_sc_, amiloride-sensitive I_sc_ reflecting ENaC-mediated sodium absorption, and cAMP-activated and CFTRinh172-sensitive I_sc_ reflecting CFTR-mediated chloride secretion did not differ between cultures from children and elderly people (**Figures 2C–G**). However, calcium-activated chloride secretion induced by apical stimulation of purinergic signaling by UTP showed an ~50% lower response in children compared to elderly people (**Figure 2H**). Transcript levels of *CFTR* as well as calcium-activated chloride channel *TMEM16A* did not differ in nasal cultures from children compared to elderly, although there was a trend toward higher expression of *TMEM16A* in the elderly group (**Figures 2I, J**). We also analyzed cellular localization of TMEM16A protein by immunostaining, which showed no expression in ciliated cells and low expression in MUC5AC^+^ cells, whereas most of the TMEM16A signal was localized to other cells that do not express MUC5AC (**Supplementary Figure 1**). To assess the role of TMEM16A in the age-dependent difference in calcium-activated chloride secretion, we determined UTP-induced chloride secretory responses in the absence and presence of the TMEM16A inhibitor Ani9 (**Figures 3A–D**). Ani9 blocked 80%–100% of the UTP-induced I_sc_ in both children and elderly (**Figure 3E**).

### Proteome Analysis of Nasal Epithelial Cultures From Children and Elderly People

**Figure 4:**
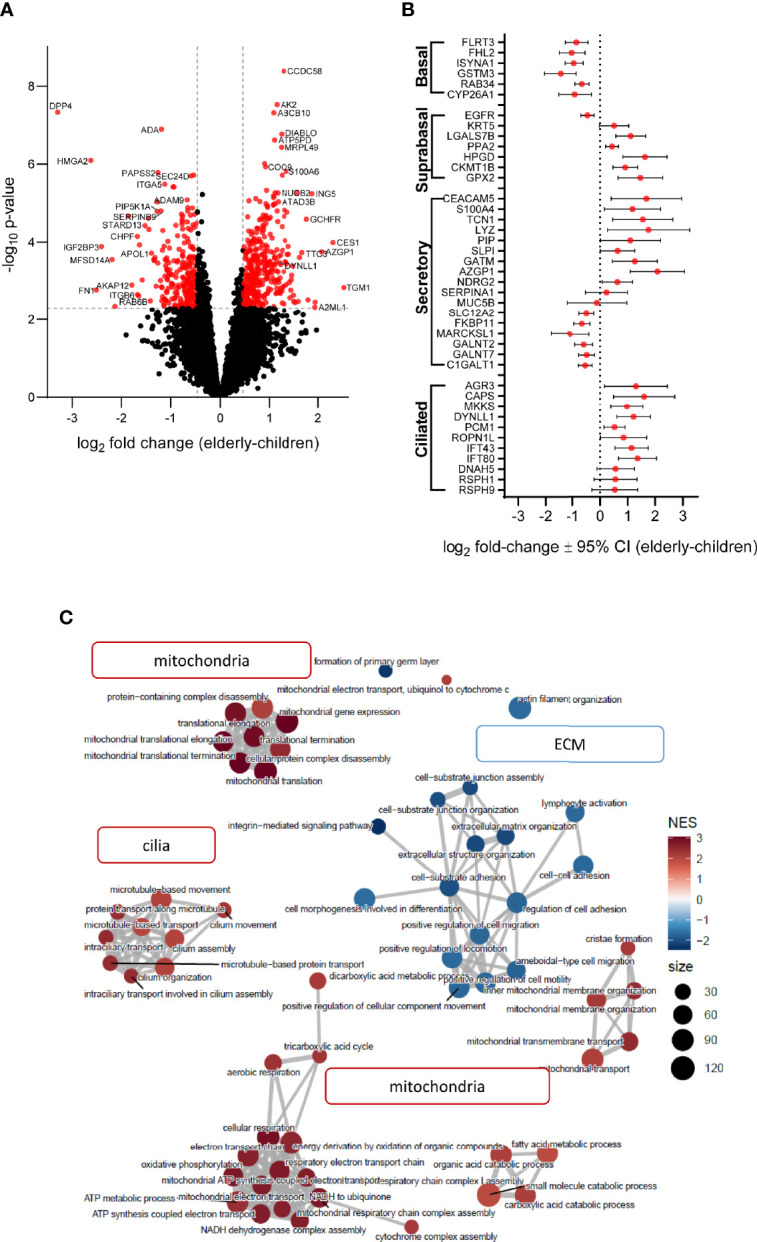
Age-related differences in proteome signatures of nasal epithelial cultures from healthy children compared to elderly people. **(A)** Differential protein expression between age groups with reference to children (volcano plot) illustrates fold-change expression (log_2_ scaling) and significance (–log_10_ scaling, adjusted *p*-value). Significantly differently abundant proteins are colored red (adjusted *p*-value < 0.05 and fold-change > 1.38). **(B)** Protein expression of airway cell subtype markers. The log_2_ fold-change with 95% confidence interval (CI) of detected proteins for each marker is plotted on the *x*-axis. **(C)** Enrichment map of top 60 gene ontology/biological process terms yielded by GSEA. Each node corresponds to a gene set with either high (red) or low (blue) normalized enrichment score (NES) in the elderly group. Node size correlates with number of genes that are annotated to the term.

Next, we assessed age-related proteome profiles in nasal epithelial cultures from children and elderly people by liquid chromatography tandem mass spectrometry. Overall, 7,073 proteins were detected. Principal component analysis confirmed absence of outliers and showed separation of samples according to age already using the full proteome, indicating that age is a primary source of variance (**Supplementary Figure 2**). Linear modeling revealed that among the differentially expressed proteins [alpha = 0.005 (FDR < 5%), |log_2_(FC)| > median residual SD], 364 proteins were upregulated and 254 proteins were downregulated in the elderly group compared to children (**Figure 4A** and **Supplementary Table 1**). Note that increasing the stringency of feature selection to the very high level [alpha = 0.0001 (FDR < 0.7%), |log_2_(FC)| > 1] resulted in full separation of elderly group from children in the post-hoc PCA and hierarchical clustering (**Supplementary Figure 3**). Some of the most significantly upregulated proteins in elderly are involved in mitochondrial function (ABCB10, ATAD3B, ATP5PD, and MRPL49), while upregulated proteins in children were related to immune-epithelial cell interactions (DPP4 and ADA) and extracellular matrix organization (FN1, ITGA5, ITGB6, and ADAM9). To capture potential differences in epithelial cell populations between children and elderly, we analyzed the expression pattern of epithelial cell-type markers (51). Taking the top 20 significantly differentially expressed markers for each subtype, proteins associated with basal cells were overall decreased in elderly (log_2_ fold-change = −0.47 ± 0.26), accompanied by an increase in the abundance of suprabasal (log_2_ fold-change = 0.51 ± 0.19) markers. Secretory cell markers showed higher variability (“Secretory” cluster: log_2_ fold-change = −0.26 ± 0.28; “Secretory N” cluster: log_2_ fold-change = −0.04 ± 0.21, “Submucosal gland goblet cell” cluster: log_2_ fold-change = 0.02 ± 0.23), whereas genes associated with serous cells were increased in elderly samples (log_2_ fold-change = 0.39 ± 0.24). Ciliated cell markers were significantly upregulated in the elderly group (log_2_ fold-change = 0.62 ± 0.18) (**Supplementary Table 4**). To visualize protein expression patterns, a subset of known cell-type markers are showcased in **Figure 4B**. Next, we performed gene set enrichment analysis (GSEA) to understand patterns of age-dependent changes in protein expression and related biological processes. This analysis revealed an upregulation of protein sets related to mitochondria and oxidative phosphorylation, as well as cilia-related processes in the elderly. Furthermore, gene sets related to extracellular matrix were downregulated in elderly people compared to children (**Figure 4C**, **Supplementary Tables 2****,** **3** and **Supplementary Figure 4**).

## Discussion

This study provides an integrated comparison of age-related differences in the cell-type composition, mucin expression, ion transport properties, and the proteome of highly differentiated primary nasal epithelial cultures from children and elderly people. Our data show that cultures from children displayed less ciliated cells and more MUC5AC^+^ secretory cells, as well as expressed more MUC5AC (**Figure 1**), while TMEM16A-mediated chloride secretion was lower compared to cultures from elderly people (**Figures 2**, **3**). These findings were complemented by proteome analysis, which revealed age-dependent differences in protein signatures related to cilia development and mucin secretion in addition to other pathways consistent with aging (**Figure 4**). Collectively, these data provide novel insights into aging of the airway epithelium and may aid our understanding of its role in the pathogenesis of age-dependent phenotypes observed in a spectrum of acute and chronic airways diseases.

In our study, we used highly differentiated primary epithelial cultures from brushed cells collected by a non-invasive sampling technique, which enabled the comparison of healthy children and elderly people. Our morphological analysis revealed a lower number of ciliated cells and higher levels of MUC5AC^+^ secretory cells in children, suggesting that the differentiation and cell-type composition of nasal epithelial cultures are age-dependent (**Figure 1**). These observations are in line with data from a recent single-cell RNA sequencing study that showed lower number of ciliated cells and higher amount of goblet cells in nasal brushes from children vs. adults (20). In the same report, epithelial cells from children displayed a pre-activated innate response profile, which is concordant with our findings of elevated immune cell–epithelial cell interaction signatures in children vs. elderly people by proteome analysis (**Figure 4**, **Supplementary Table 3** and **Supplementary Figure 4**). Age-related changes in airway epithelial cell lineages are further supported by another study describing a higher ciliated cell-to-club cell ratio in aged mice (52). The molecular basis of enhanced ciliary differentiation with advanced age and its role in airway homeostasis remain unknown. We speculate that a higher density of ciliated cells may help to maintain effective MCC in the aging airways. When viewed in combination, these studies indicate that our *in vitro* cultures retained age-dependent *in vivo* characteristics, supporting the relevance of this model system.

Our electrophysiological studies showed an overall similar transepithelial ion transport profile of nasal epithelial cultures from children and elderly people. Specifically, we did not find evidence of age-dependent differences in basal bioelectric properties, ENaC-mediated sodium absorption, or CFTR-mediated anion secretion across the nasal epithelium (**Figure 2**). However, we found selective upregulation of UTP-induced chloride secretory responses in the elderly group (**Figures 2**, **3**). A direct role of TMEM16A was supported by pharmacological inhibition of UTP-responses by the TMEM16A inhibitor Ani9 (**Figure 3**) (53, 54). These data suggest that calcium-activated chloride/fluid secretion and airway surface liquid regulation *via* TMEM16A may be more relevant in the airways at older age. However, the functional relevance of this finding needs to be tested in future studies.

Our proteome analysis provided independent evidence of substantial age-dependent differences in airway epithelial structure and function in children compared to elderly people. Consistent with our morphological analysis, an upregulation of ciliated cell markers (AGR3 and CAPS), axoneme components (DYNLL1, IFT43, and IFT80), and enrichment of cilia-related pathways was characteristic of the epithelial proteome of elderly people (**Figures 1**, **4**) (55). We also observed a decreased expression of basal cell markers in the elderly proteome, suggesting cellular senescence, a well-known hallmark of aging (56, 57). Furthermore, we found significantly increased expression of the cyclin-dependent kinase inhibitor CDKN1B (p27) in elderly, a known inducer of the senescence cell cycle arrest (58). While MUC5AC was below the detection limit in the proteome analysis, we found an upregulation of proteins related to mucin glycosylation (C1GALT1, GALNT2, and GALNT7) and mucin secretion (SLC12A2 and IL1R1) in children, in agreement with the observed increase of MUC5AC by Western blot (59, 60). Consistent with enhanced MUC5AC production, we also observed increased type-2 inflammatory response signature in children (**Supplementary Figure 4**). While mucin-related genes were elevated, markers associated with serous cells (LYZ and PIP) and anti-microbial secreted proteins (SLPI and CLU) had decreased levels in children (**Supplementary Figure 4**). To the best of our knowledge, changes in MUC5AC expression have not been reported in the context of aging. However, this finding may contribute to the age-dependent predisposition to certain muco-obstructive diseases, such as allergen-induced asthma in children where MUC5AC has been implicated in the pathogenesis of asthma severity, compared to COPD in elderly patients that is characterized predominately by an increase in MUC5B (61–63). Interestingly, we observed a strong increase in mitochondria-related proteins and pathways in nasal cultures from elderly people. While higher abundance of ciliated cells in the elderly may be associated with higher energy expenditure (64), we also found enrichment of glycolytic pathways in children (**Supplementary Figure 4**), which may suggest a shift towards oxidative phosphorylation with aging, which would be similar to metabolic changes described in the aging brain and muscle tissue (65, 66). Increased mitochondrial mass and mitochondrial dysfunction, accompanied by increased generation of reactive oxygen species (ROS), are also markers of cellular senescence (67, 68). We found that not only mitochondrial proteins were upregulated in the elderly, but also a number of enzymes with antioxidant functions were increased, including the mitochondrial superoxide dismutase SOD1 (**Supplementary Figure 4**), suggesting an adaptive response to dampen the oxidative phenotype of cellular senescence and aging (69, 70). While we observed markers associated with increased cellular senescence in the elderly as compared to children, future studies should determine its role in the aging epithelium.

Although we could link our morphological observations with proteome data, we did not find evidence for differences in purinergic receptor activation or calcium signaling pathways that may explain our functional findings. This is likely related to the low abundance of regulatory proteins in those signaling cascades as well as the limitations of the global proteomics approach to capture post-translational modifications that govern many signaling events (71). However, mitochondria are known to play a role in the compartmentalization of calcium signals upon P2Y2-receptor stimulation in airway epithelial cells, by acting as a calcium-buffering system (72). It is possible that aging-associated mitochondrial dysfunction may disrupt the spatiotemporal fine-tuning of intracellular calcium levels, leading to enhanced calcium-activated chloride secretion by TMEM16A.

In summary, this is the first study describing age-dependent structural and functional differences in highly differentiated human primary nasal epithelial cultures, including an in-depth comparison by proteome analysis. We observed lower abundance of ciliated cells and higher expression of MUC5AC in children vs. elderly people, which correlated with age-dependent proteome signatures. Ion transport studies showed overall similarities in ENaC and CFTR function, with lower TMEM16A-mediated chloride secretion in children. These data indicate intrinsic, age-related phenotypic differences in the airway epithelium, which may help to better understand the effect of aging on innate mucosal defense and age-dependent airways disease phenotypes. Further work is needed to identify the underlying mechanisms and clinical relevance of these findings.

## Data Availability Statement

The datasets presented in this study can be found in online repositories. The names of the repository/repositories and accession number(s) can be found below: ProteomeXchange, accession no: PXD030130

## Ethics Statement

The studies involving human participants were reviewed and approved by the ethics committee at the Charité-Universitätsmedizin Berlin (EA2/066/20). Written informed consent to participate in this study was provided by the participants’ legal guardian/next of kin.

## Author Contributions

Conception and design of the study: AB, PM-B, and MaM. Acquisition, analysis, and interpretation of data: all authors. Drafting the article or revising it critically for important intellectual content: all authors. All authors contributed to the article and approved the submitted version.

## Funding

This study was supported by grants from the German Federal Ministry of Education and Research (RECAST 01IK20337, NUM-COVID 19 Organo-Strat 01KX2021, and 82DZL009B1) and the German Research Foundation (CRC 1449–431232613 A01 and Z02 and project no. 450557679).

## Conflict of Interest

The authors declare that the research was conducted in the absence of any commercial or financial relationships that could be construed as a potential conflict of interest.

## Publisher’s Note

All claims expressed in this article are solely those of the authors and do not necessarily represent those of their affiliated organizations, or those of the publisher, the editors and the reviewers. Any product that may be evaluated in this article, or claim that may be made by its manufacturer, is not guaranteed or endorsed by the publisher.
