# Supplementary material for: Age-Related Differences in Structure and Function of Nasal Epithelial Cultures From Healthy Children and Elderly People
Source: Front Immunol. 2022 Feb 28;13:822437. doi: 10.3389/fimmu.2022.822437 (PMC8918506; doi:10.3389/fimmu.2022.822437)
Supplement: Supplementary file 1 [file DataSheet_1.docx]

**Supplementary Figure 1. Principal component analysis (PCA) of total data matrix.**


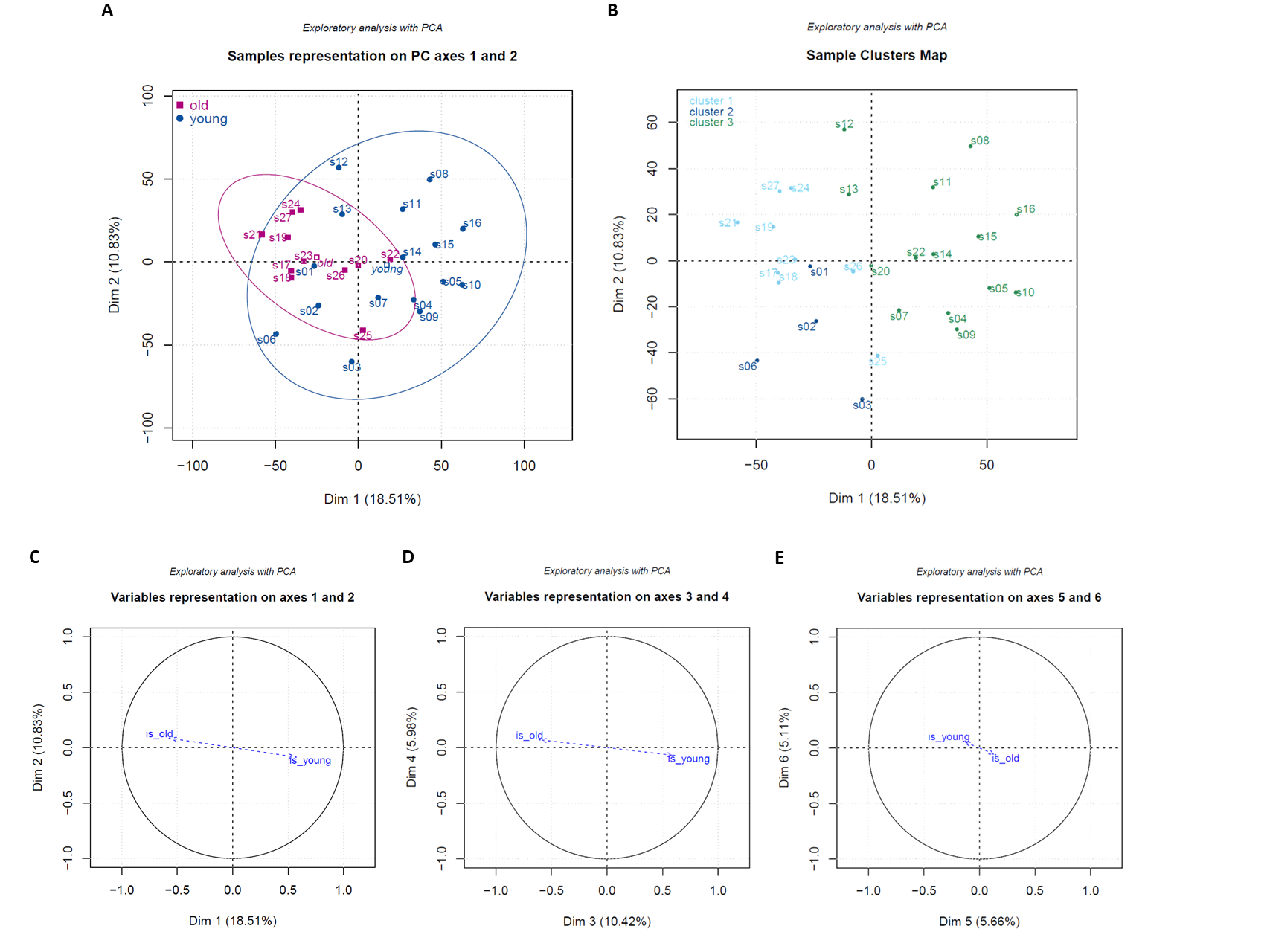


PCA (carried out using FactoMineR package (Le et. al 2008)) showed that the first 6 principal components (PC) were delivering minimal mean error, thus presenting the most informative part of the data and were explaining ~ 56% of total variance. **(A)** PCA score plot of nasal epithelial cultures from children and elderly people, each point represents an individual (n = 16 and 11 individuals per group). Full set of 7073 proteins was used. **(B)** K-means partitioning of samples into three clusters using matrix of first six principal components. Results, presented in the first two principal components, show that cluster 1 consists entirely of samples from elderly people, while cluster 2 and cluster 3 entirely of samples from children with only two misclassifications – samples s20 and s22 from elderly patients were allocated to young group cluster 3. **(C) – (E)** PCA loading plots in the first two PC **(C)**, PC3 and PC4 **(D)** and PC5 and PC6 **(E)**, reveal that factor age has significant contribution to the PC1 and PC3.
