# Supplementary material for: Age-Related Differences in Structure and Function of Nasal Epithelial Cultures From Healthy Children and Elderly People
Source: Front Immunol. 2022 Feb 28;13:822437. doi: 10.3389/fimmu.2022.822437 (PMC8918506; doi:10.3389/fimmu.2022.822437)
Supplement: Supplementary file 2 [file DataSheet_2.docx]

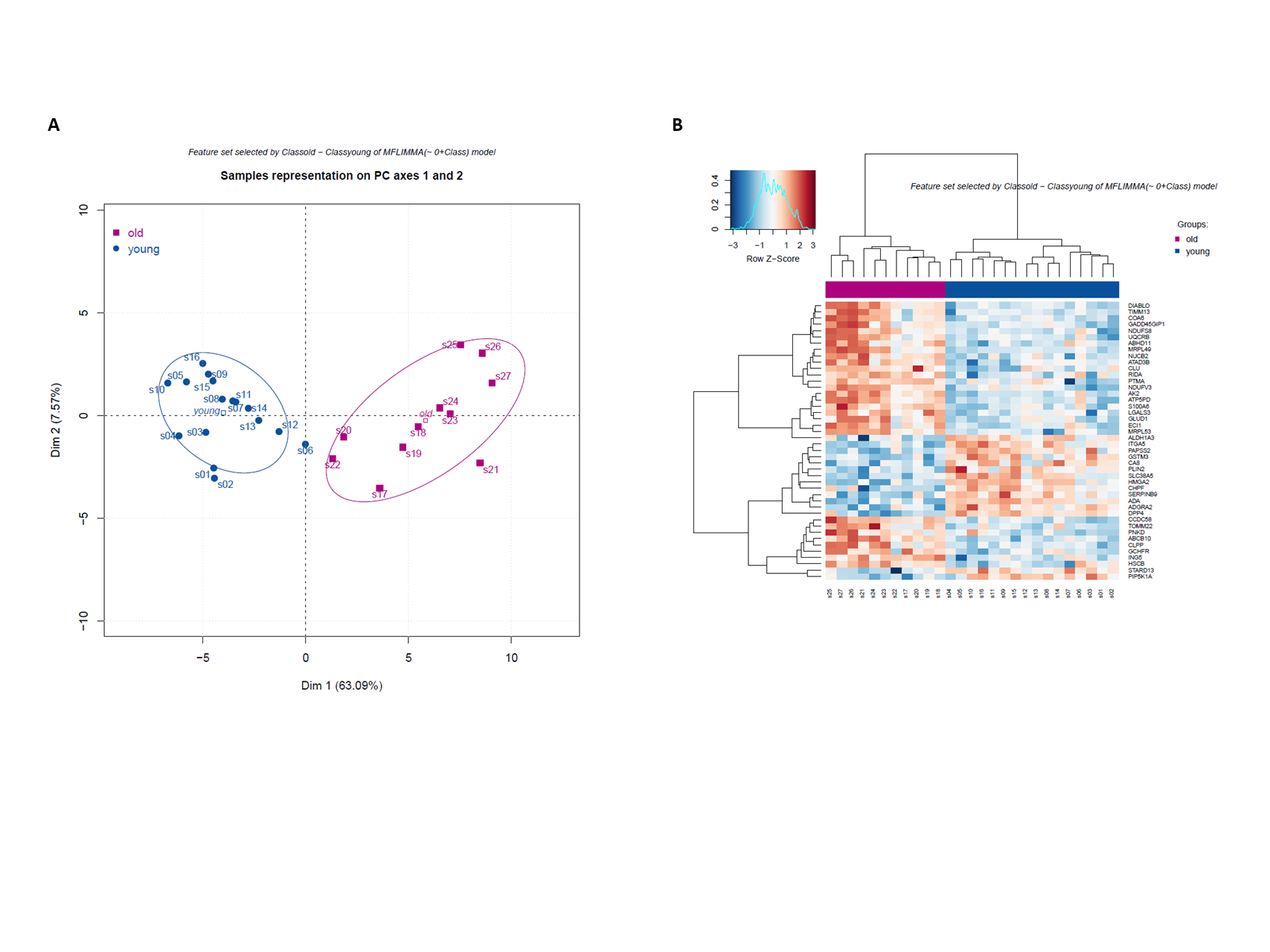


**Supplementary Figure 2. Post-hoc analysis of top 44 ultra-strong regulated features (selected using alpha = 0.0001 and |logFC|> 1). (A)** PCA score plot of nasal epithelial cultures from children and elderly people. **(B)** Hierarchical clustering of nasal epithelial cultures from children and elderly people using top 44 most regulated proteins.
