# Supplementary material for: Age-Related Differences in Structure and Function of Nasal Epithelial Cultures From Healthy Children and Elderly People
Source: Front Immunol. 2022 Feb 28;13:822437. doi: 10.3389/fimmu.2022.822437 (PMC8918506; doi:10.3389/fimmu.2022.822437)
Supplement: Supplementary file 3 [file DataSheet_3.docx]

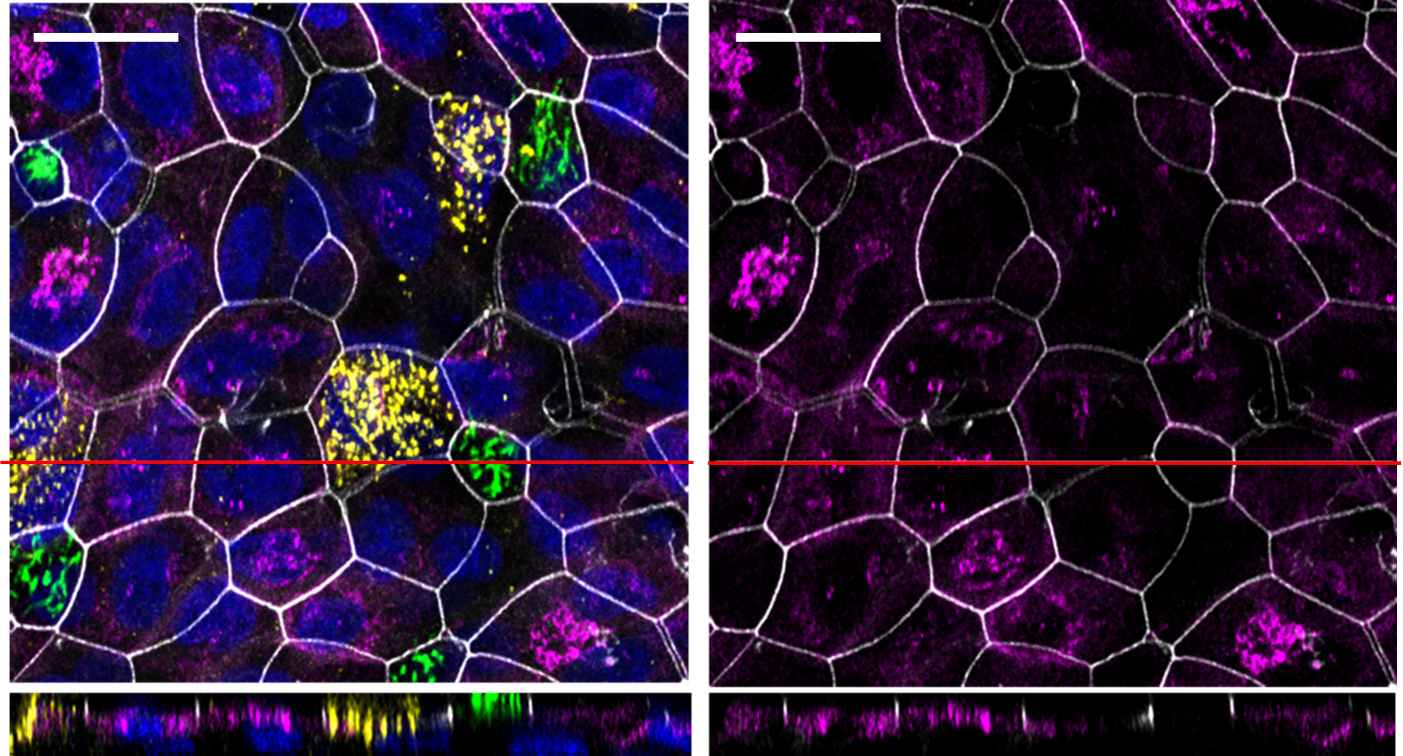


**Supplementary Figure 3. Representative images of TMEM16A immunolocalization in primary nasal epithelial cultures.**

Magenta: TMEM16A, white: ZO-1 (tight junctions), green: α-tubulin (ciliated cells), yellow: MUC5AC (secretory cells) and blue: Hoechst (cell nuclei). Left panel represents an overlay of all confocal channels, right panel represents an overlay of ZO-1 and TMEM16A. Stacks were acquired with 63X immersion objective in confocal mode with Lightning module and maximal Z-projections with Z-sections at indicated red lines are shown. Scale bar 20 µm.
