# Supplementary material for: Age-Related Differences in Structure and Function of Nasal Epithelial Cultures From Healthy Children and Elderly People
Source: Front Immunol. 2022 Feb 28;13:822437. doi: 10.3389/fimmu.2022.822437 (PMC8918506; doi:10.3389/fimmu.2022.822437)
Supplement: Supplementary file 4 [file DataSheet_4.docx]

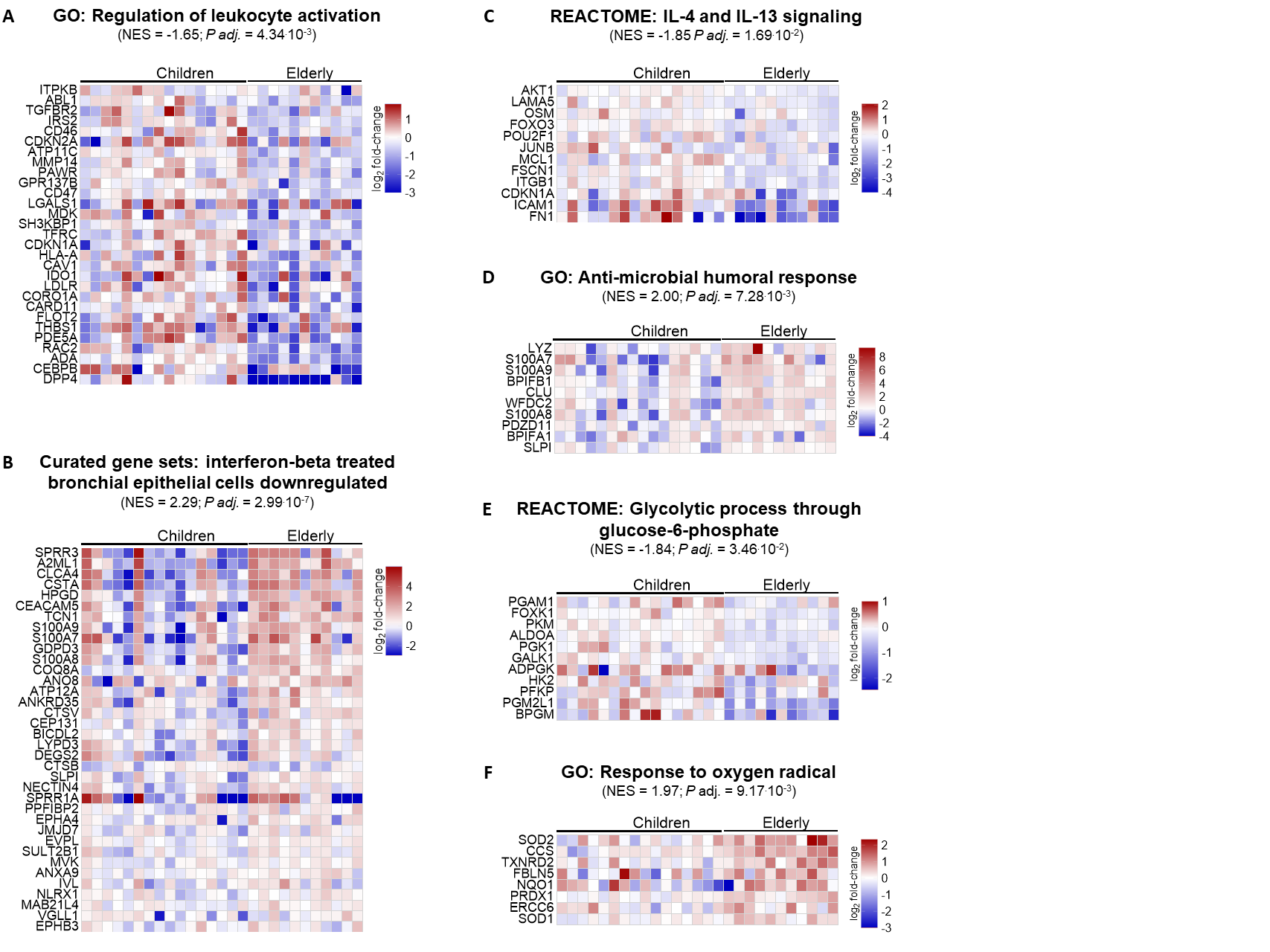


**Supplementary Figure 4. GSEA pathways for differentially expressed proteins in nasal epithelial cultures from healthy children compared to elderly people (A-F).**

Heatmaps highlighting pathways related to **(A)** leukocyte activation, **(B)** interferon-beta signaling, **(C)** type-2 inflammatory signaling, **(D)** anti-microbial humoral response, **(E)** glycolysis, and **(F)** response to oxygen radicals. Mean gene expression value of leading-edge genes of each gene set is plotted, each column represents the expression data derived from samples from different individuals.
